# Regulators mount up: the metabolic roles of apoptotic proteins

**DOI:** 10.3389/fceld.2023.1223926

**Published:** 2023-07-03

**Authors:** James H. Schofield, Zachary T. Schafer

**Affiliations:** Department of Biological Sciences, University of Notre Dame, Notre Dame, IN, United States

**Keywords:** apoptosis, metabolism, caspases, mitochondria, MOMP

## Abstract

The induction of apoptosis, a programmed cell death pathway governed by activation of caspases, can result in fundamental changes in metabolism that either facilitate or restrict the execution of cell death. In addition, metabolic adaptations can significantly impact whether cells in fact initiate the apoptotic cascade. In this mini-review, we will highlight and discuss the interconnectedness of apoptotic regulation and metabolic alterations, two biological outcomes whose regulators are intertwined.

## Introduction

Apoptosis is a programmed cellular suicide mechanism with important roles in the maintenance of multicellular organisms. The classical intrinsic apoptotic pathway begins with an apoptotic stimulus that alters pro-apoptotic proteins at the mitochondria. Subsequently, this leads to outer membrane permeabilization (MOMP) as a consequence of Bax/Bak oligomerization (Oltval et al., 1993; Shi et al., 2003; Buytaert et al., 2006; McArthur et al., 2018). MOMP allows for the secretion of soluble cytochrome *c* into the cytosol where it binds to apoptotic protease activating factor 1 (Apaf-1) and causes the formation of the apoptosome (Schafer and Kornbluth, 2006; Riedl and Salvesen, 2007). Apoptosome formation triggers the concomitant cleavage and activation of caspase 9, which cleaves and activates caspases 3 and 7 ultimately culminating in the death of the cell (Xue and Robert Horvitz, 1995; Elmore, 2007; McIlwain et al., 2013). Each of the proteins listed are critically important for proper execution of the intrinsic apoptosis pathway. However, these proteins (and others known to regulate apoptosis) do not solely function as determinants of whether a cell lives or dies. Rather, many of the main players in apoptotic pathways have other, oftentimes vital, roles in cellular processes such as metabolism. Perhaps the best example of the dual role of certain apoptotic proteins within metabolic pathways is the aforementioned cytochrome *c*, whose apoptotic role was discovered in the laboratory of Xiaodong Wang (Liu et al., 1996; Li et al., 1997). While the discovery of a pro-apoptotic role for a protein best understood to function in the mitochondrial respiratory chain was (at the time) surprising, the capacity of cytosolic cytochrome *c* to activate caspases is now central to our understanding of programmed cell death. In this article, it is our objective to highlight the sometimes-underappreciated metabolic functions of apoptotic regulators and to discuss the circumstances in which metabolism directly impinges upon the execution of apoptosis.

### Glucose flux and the capacity of p53 to induce apoptosis

As mentioned above, turnover of aged or defective cells in the human body can often occur through the induction of apoptosis (Kerr et al., 1972; Fawthrop et al., 1991). The execution of apoptosis is tightly regulated by a number highly conserved and distinct signal transduction networks. In addition, proteins which regulate apoptotic death can be broadly separated into two main categories: pro-apoptotic and anti-apoptotic. Anti-apoptotic proteins function to provide defenses against the activation of proteases that execute the apoptotic program. Meanwhile, pro-apoptotic effectors can sense deleterious events in the cell and sound the alarm to initiate the induction of apoptosis. Alternatively, pro-apoptotic effectors can function directly in the cleavage of target proteins that ultimately contribute to the orderly dismantling of the cell. Dysregulation of these coordinated pathways is linked to pathological conditions like fibrosis, autoimmune disease, neurodegeneration, and cancer (Elmore, 2007).

One of the most well-studied pro-apoptotic regulators is p53, a known tumor suppressor that integrates extracellular and intracellular signals to promote cell death (Surget et al., 2013; Aubrey et al., 2018). The best characterized activator of p53 is DNA damage, which ultimately causes phosphorylation and stabilization of the p53 protein and thus allows is to transcriptionally activate downstream targets. However, nutrient availability and energy demands can also impact p53 activity (Horn and Vousden, 2007). Previous work has demonstrated that high levels of ADP promote the binding of p53 to DNA whereas elevated ATP levels block this interaction (Okorokov and Milner, 1999). There are also significant links between the withdrawal of growth factors, changes in nutrient uptake and p53. For example, serum starvation can often promote p53-mediated increased apoptosis due to elevated expression of p53 upregulated modulator of apoptosis (PUMA) (Ekoff et al., 2007). In contrast, if glucose uptake is maintained (e.g., through localization of Glut1 to the plasma membrane) after growth factor withdrawal, apoptosis is attenuated as a consequence of glycolytic flux-mediated inhibition of p53 and PUMA (Zhao et al., 2008).

In addition to the links between glucose metabolism and PUMA, the withdrawal of extracellular glucose from cells acts as a trigger for the nucleocytoplasmic isoform of the metabolic enzyme malate dehydrogenase (Lee et al., 2009). This enzyme is normally involved in the malate-aspartate shuttle, and can function to stabilize and transactivate p53 to promote p53-dependent cell-cycle arrest. Similarly, withdrawal of glucose often leads to the activation of the AMP-activating protein kinase (AMPK), a cell cycle arrest at the G (1)/S stage, and the induction of p53 (Jones et al., 2005; Okoshi et al., 2008).

Oftentimes, the regulation of apoptosis by p53 is largely a consequence of post-translational modifications to key residues that affect protein stability and ultimately the genes targeted by this transcription factor. Indeed, the AMPK-mediated p53 activation during glucose deprivation is dependent upon the phosphorylation of serine 15 (Jones et al., 2005). Similarly, the capacity of glucose to prevent p53 induction of PUMA depends on a decrease in the phosphorylation of serine 46 (Garufi and D’Orazi, 2014). In addition to these roles, the decrease of glucose utilization can lower the apoptotic threshold of cells due to alterations in the cytoplasmic function of p53. A good example of this p53-mediated effect can be found in patient-derived models of glioblastoma which have been pharmacologically restricted in their glucose uptake. These tumors are primed for apoptosis *in vivo* but are kept alive owing to the sequestration of cytoplasmic p53 by Bcl-xL and the prevention of p53 mediated transcription of pro-apoptotic factors keeping the tumors just below the threshold of cell death (Mai et al., 2017). Lastly, p53 can function to ensure that elevated glucose flux cannot prevent the initiation of the apoptotic cascade by inhibiting the expression of the glucose transporters Glut1 and Glut4 (Schwartzenberg-Bar-Yoseph et al., 2004).

### TIGAR: a protein that functions downstream of p53 to impact metabolism

The understanding of how metabolic flux impacts p53 regulation of apoptosis also inspired efforts to study downstream effectors of p53 that impact metabolism. One particular example of p53-mediated metabolic regulators is TP53-inducible glycolysis and apoptosis regulator (TIGAR). TIGAR was discovered through microarray analysis of genes following p53 induction and functions to attenuate glycolytic flux through regulation of fructose-2,6-bisphosphatase (Bensaad et al., 2006). The capacity to regulate fructose-2,6-bisphosphatase is dependent on TIGAR’s phosphatase activity, which inhibits the flow of carbon units through glycolysis and prevents glycolysis from counteracting the action of p53. In addition, the activity of TIGAR not only blocks glycolysis, but also can promote cell survival by diverting glycolytic flux into biosynthetic or of antioxidant-generating pathways. One key determinant of TIGAR function is subcellular localization, which is highly dependent on various stress stimuli encountered by the cell. While in the cytoplasm, TIGAR antagonizes glycolysis and shifts carbon flux into the pentose phosphate pathway (PPP), which promotes production of NADPH for redox homeostasis or nucleotide synthesis. However, exposure to DNA damaging chemotherapeutic agents causes TIGAR to translocate to the nucleus where it halts the cell cycle and promotes DNA damage repair in a p53-dependent manner (Yu et al., 2015). Hypoxia can also impact the subcellular localization of TIGAR. Under hypoxic conditions, TIGAR can localize to the outer mitochondrial membrane where it binds to hexokinase 2 (HK2) and increases HK2 activity. The binding of TIGAR to HK2 increases glycolytic flux and helps to lower mitochondrial reactive oxygen species (ROS) with TIGAR functioning as a scaffold for HK2 activation (Cheung et al., 2012). Studies have also examined the loss of TIGAR, which can promote glucose oxidation and glycolysis in myocardial tissue demonstrating the importance this enzyme plays in throttling glycolytic flux following p53 activation (Okawa et al., 2019). Taking all of this together, it is clear that TIGAR is an important link between p53 and glycolytic metabolism. However, p53 induced genes can reciprocally counteract this effect to facilitate p53-mediated apoptosis. Thus, p53 exemplifies a “push-pull” dynamic between one of the best studied tumor suppressors (p53) and a common metabolic alteration observed in cancer cells (increased glycolysis) with important ramifications for disease progression.

### MOMP and metabolism

Following the initiation of apoptotic signaling, pores are formed in the outer mitochondrial membrane during MOMP to allow proteins from the mitochondrial intermembrane space, including cytochrome *c*, to reach the cytosol and propagate the apoptotic cascade (Westphal et al., 2011). Although cytochrome *c* release was once considered the point of no return for cell death, research over the past decade indicates that sub-lethal amounts of MOMP may occur in response to certain apoptotic stimuli. This phenomenon is known as incomplete or “minority” MOMP (Tait et al., 2010; Ichim et al., 2015). For a cell to survive MOMP it must either limit the formation of the pore, neutralize the cytochrome *c* that is released, or functionally adapt to improve its fitness in this environment.

Blocking formation of the pore in the mitochondrial membrane is a logical place for the beginning of our discussion on links between metabolism and MOMP. Indeed, bioactive lipids, such as sphingolipids, have been implicated as a significant player in the regulation of MOMP in a variety of cell types. More specifically, ceramide, a class of sphingolipid, can induce apoptosis through a mechanism that is dependent on the molecular machinery required to promote MOMP (Taha et al., 2006; von Haefen et al., 2002). Furthermore, inhibition of sphingolipid metabolism through the use of pharmacological inhibitors is capable of preventing MOMP as it blocks the interaction between ceramide metabolites and inducers of MOMP (Chipuk et al., 2012). Relatedly, cancer cells can manipulate sphingolipid metabolism to decrease the intracellular levels of ceramide to maintain mitochondrial membrane integrity. As an example, sphingomyelin synthases are activated in leukemic cells to decrease the levels of ceramide and prevent stress-induced apoptosis (Dolgachev et al., 2004; Separovic et al., 2007; Lafont et al., 2010).

However, if a cell cannot alter metabolism to prevent MOMP, another strategy to survive involves the neutralization of the cytochrome *c* that is released from the inner membrane space. One such strategy to neutralize the execution of apoptosis employed by these cells is the rewiring of metabolism to defang cytosolic cytochrome *c* and conserve viability. Retroviral cDNA screens following MOMP have shown a role for glyceraldehyde-3-phosphate dehydrogenase (GAPDH) in promoting survival through increased glycolytic flux in the cell when caspase activation was inhibited (Colell et al., 2007). Interestingly, cytochrome *c* released from these cells following MOMP was re-localized back to the mitochondria only in cells with increased GAPDH expression. These interesting results indicate that GAPDH can promote recovery and function of those mitochondria post-MOMP. In addition, the pro-apoptotic activity of cytochrome *c* relies on its redox state and is known to be controlled by ROS signaling. Increased glucose flux into the PPP of cancer cells generates elevated levels of the antioxidant glutathione, which can also function to antagonize cell death by numerous mechanisms [e.g., ferroptosis (Dixon et al., 2012)]. However, with regards to apoptotic regulation, glutathione can inactivate cytochrome *c* following MOMP to prevents apoptosis (Vaughn and Deshmukh, 2008). Instead of attempting to inactivate cytochrome *c*, a cell can also survive during MOMP by simply eliminating the cytochrome c that is released. RNAi screens in both neurons and cancer cells uncovered a conserved strategy for dealing with cytoplasmic cytochrome *c*. p53-associated Parkin-like cytoplasmic protein (PARC) functions as an E3 ligase that targets cytochrome *c* for destruction following mitochondrial stress and minority MOMP to promote viability (Gama et al., 2014). In some cases, adaptation to a sub-lethal MOMP benefits a cell and may eventually promote tumorigenesis. Due to the fact that minority MOMP induces only limited caspase activation to cause DNA damage, this can contribute to genomic instability and result in oncogenic transformation (Ichim et al., 2015). Malignant cells that have been treated with agents to induce MOMP allows for cytosolic cytochrome c to active the heme-regulated inhibitor kinase (HRI) engaging the integrated stress response (ISR) (Kalkavan et al., 2022). The engagement of the ISR by cancerous cells generates a drug-resistant cell population that is not only protected against apoptosis but also abrogates the efficacy of the therapy. Therefore, minority MOMP can not only promote tumorigenesis but also contribute to therapeutic resistance in malignant cells.

### Metabolic functions of Bcl-2 family members

The formation of pores in the outer mitochondrial membrane as a consequence of the regulation of Bcl-2 family members is often critical to the induction of apoptosis. As discussed above, the Bcl-2 proteins have multiple structural and functional similarities with the ability to be either pro- or anti-apoptotic. For example, Bcl-2 family members are well known to contain BH3 motifs (Bcl-2 homology 3) (Blaineau and Aouacheria, 2009), which have diverse biological functions in the regulation of apoptosis. However, many of the Bcl-2 family members also have a significant role in the regulation of metabolism. Notably, while Bcl-2 family members have been shown to regulate mitochondrial dynamics and thereby indirectly alter metabolic pathway utilization (Autret and Martin, 2010)), we have chosen to focus the section below on the involvement of Bcl-2 family members on metabolic flux directly.

One such Bcl-2 family member with a significant metabolic function is Myeloid cell leukemia-1 (MCL1 or Mcl-1) which localizes to the mitochondria to prevent MOMP and blocks apoptosis (Kozopas et al., 1993; Reynolds et al., 1994; Zhou et al., 1997; Opferman et al., 2003; Opferman et al., 2005). Mcl-1 is among the most overexpressed survival proteins across all human cancers and is linked to poor survival and therapeutic resistance (Wuillème-Toumi et al., 2005; Wei et al., 2006; Beroukhim et al., 2010). In addition to its anti-apoptotic function, Mcl-1 has been shown to facilitate nutrient recycling through the induction of mitophagy. Although Mcl-1 inhibits nonselective autophagy caused by nutrient starvation, Mcl-1 promotes the targeted destruction of depolarized mitochondria ensuring a functional mitochondrial pool to meet energetic needs (Moyzis et al., 2022). However, increased mitophagy is not always beneficial to the cell. Recent investigations have suggested that overactive mitophagy may prove detrimental to the cell and compromise viability depending on the context of the cellular stressor (Hawk et al., 2018). One can therefore postulate that perhaps a sustained increase in Mcl-1 activity would deplete the mitochondrial pool below required levels.

**FIGURE 1:**
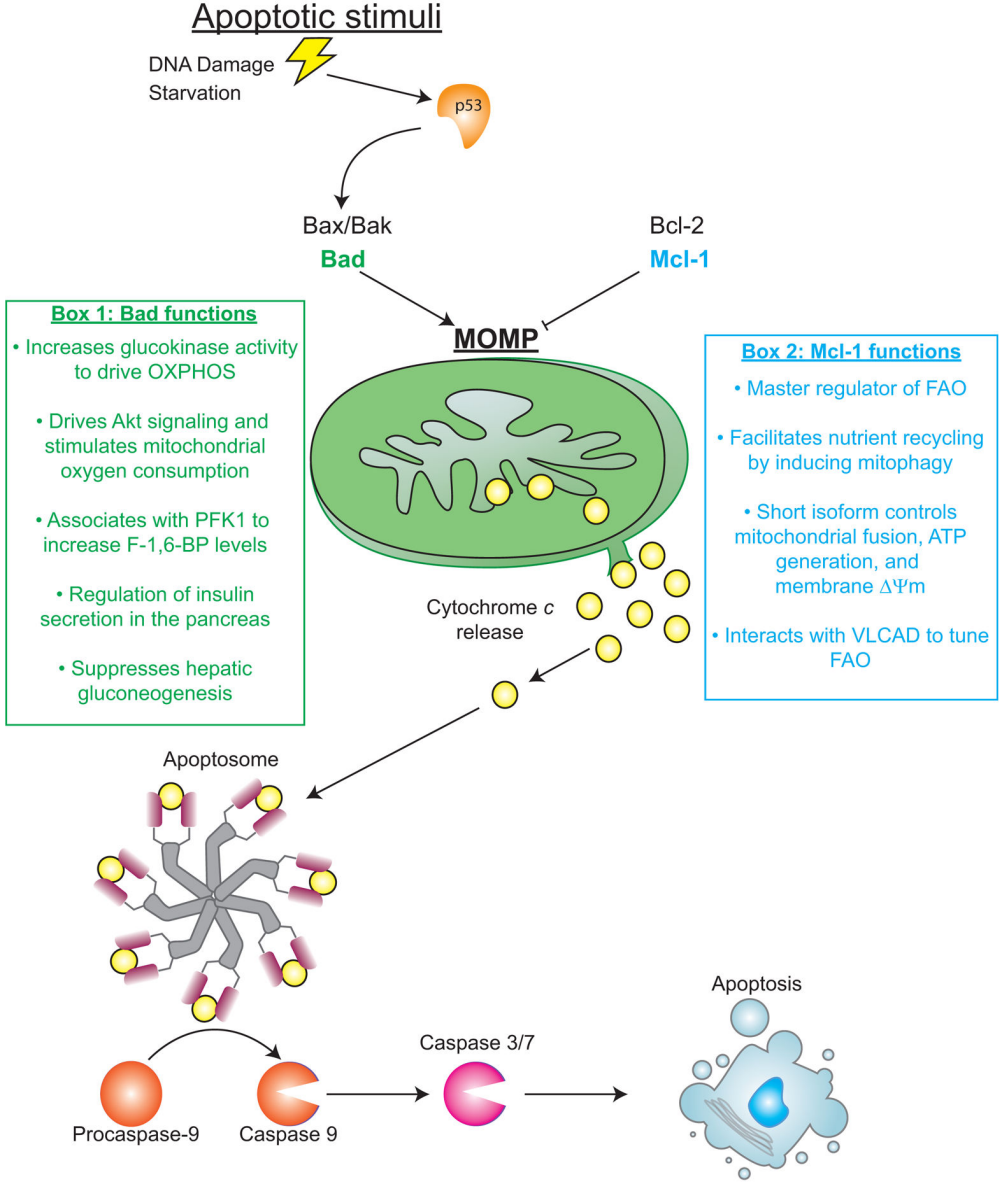
Metabolic function of apoptotic regulators.

Mcl-1 also helps cancerous cells meet energetic needs through direct regulation of metabolic pathways (see Box 2 in Figure 1). These functions can be tied to alternative splicing as distinct Mcl-1 spliceoforms can impact metabolism in unique manners. The full-length isoform associates with the outer mitochondrial membrane where it regulates cell death. However, the short isoform of Mcl-1 is imported into the inner mitochondrial matrix where it regulates fusion, ATP production, membrane potential, and maintenance of ATP synthase to support the adenylate energy charge of the cell (Perciavalle et al., 2012). The short isoform of Mcl-1 also has a direct impact on fatty acid oxidation (FAO). The *α* helix of Mcl-1 directly interacts with very long-chain acyl-CoA dehydrogenase (VLCAD) in nutrient deprived conditions to dynamically tune FAO (Escudero et al., 2018). This metabolic action of Mcl-1 during periods of stress may provide additional support to cancer cells that extend beyond survival. In fact, in B Cell acute lymphoblastic leukemias (B-ALL), where Mcl-1 overexpression is a defining characteristic, FAO is a critical fuel source. Expression of Mcl-1 correlates with elevated FAO gene signatures in these malignancies and loss of Mcl-1 rewires fuel utilization from catabolism of fatty acids to a reliance upon glycolysis (Prew et al., 2022). As such, in the absence of apoptotic stimuli, Mcl-1 can functions as a significant regulator of the FAO program.

Pro-apoptotic Bcl-2 family members also have well-defined functions in metabolic pathways. For example, Bcl-2 associated agonist of cell death (BAD) promotes programmed cell death by binding to Bcl-2 and preventing its ability to block MOMP and cytochrome *c* release (Yang et al., 1995). BAD is often regulated by phosphorylation which determines not only its role in apoptosis, but also the specific function it plays in metabolism (See Box 1 in Figure 1). For example, BAD affects glucose utilization by forming a complex containing glucokinase at the mitochondria due to its phosphorylation at S112. As a consequence, the activity of glucokinase is increased in a fashion that drives mitochondrial oxidative phosphorylation (Danial et al., 2003). Meanwhile, phosphorylation of BAD at S118 and S99 has been shown to affect metabolism due to altered Akt signaling, stimulation of complex I and elevated mitochondrial oxygen consumption (Mann et al., 2019). BAD can also bind to other metabolic enzymes as immunoprecipitation experiments have demonstrated that it can associate with phosphofructokinase 1 (PFK1). Functional assays demonstrate that JNK1-mediated phosphorylation of BAD at T201 increases PFK1 function to raise fructose-1,6-bisphosphate generation, a key rate-limiting step of glycolysis (Deng et al., 2008). Some of the post-translational modifications to BAD are cell type-specific for distinct tissue metabolic functions. For example, modification to the S155 site in the BH3 domain of BAD allows the protein to control glucose stimulated insulin secretion in the beta cells of the pancreas (Danial et al., 2008). Like the pancreas, liver-specific activity of BAD modulates the metabolism of hepatic cells. The liver must precisely balance gluconeogenesis, the generation of glucose from other metabolic substrates, and glycolysis to maintain homeostasis. BAD maintains this physiological balance by employing phosphorylation of its BH3 domain as a sensor to activate glucokinase and suppress gluconeogenesis to properly coordinate hepatic glucose output (Giménez-Cassina et al., 2014).

### Links between dietary metabolism, apoptosis, and disease

The interplay between metabolism and apoptosis extends beyond the individual cell as whole-organism metabolism impacts death across tissue systems. For example, it has long been known that heightened availability of the sugar glucose is beneficial to tumorigenesis and cancer cell survival (Warburg, 1956; Liberti and Locasale, 2016). In fact, the metabolic rewiring of cells to promote increased glucose uptake and glycolytic flux is a hallmark of cancer (Hanahan and Weinberg, 2011). Monosaccharides in general are not universal in their impact on cell survival *versus* death, and thus, the specific form of sugar taken up by cellscould alter the balance between tumor promotion and tumor suppression.

In addition to glucose, fructose has also been shown to promote cancer through inhibition of apoptotic pathways. Nutrients taken in through diet enter the tissue of mammals at the epithelium of the small intestine and colon. In the small intestine, sugars from the diet (e.g., glucose and fructose) enter the cell through the Glut family of transporters where they are phosphorylated by hexokinase (HK) and ketohexokinase (KHK) respectively (Miller et al., 1956; Roberts and Miyamoto, 2015; Jang et al., 2018). The uptake of these metabolites is governed by the villus structure within the small intestine. Intestinal villus length is balanced by proliferation and death of intestinal epithelial cells (IECs) (Hall et al., 1994). The migration of IECs up the villus separates them from their blood supply leading to hypoxia which ultimately can cause their apoptotic death. However, fructose increases the survival of hypoxic cells as well as IECs of the small intestine in mice (Taylor et al., 2021). Fructose in the IECs results in the accumulation of fructose-1-phosphate which competes with fructose-1,6-bisphosphate for the binding pocket of the glycolytic enzyme pyruvate kinase (PKM2). In this setting, inhibition of glycolytic flux, through decreased PKM2 activity, allows for increased upstream glycolytic metabolites to counteract the hypoxic insult and thereby dampen the apoptotic stimulus (Luo et al., 2011; Taylor et al., 2021). The cancer-causing potential of fructose is solidified by the finding that excessive levels of fructose in the colon, owing to the consumption of high fructose corn syrup, increases tumorigenesis in murine models (Goncalves et al., 2019). Importantly in this study, fructose was found to saturate the Glut receptors on the IECs causing fructose levels to still be high even into the colon where it could exert its neoplastic potential. Both glucose and fructose benefit malignant cells by promoting growth while simultaneously blunting apoptotic signaling.

Alternatively, other sugar molecules—including those deemed “rare” sugars—are deleterious to cancer cells. Rare monosaccharides with structural similarity to glucose or fructose induce apoptosis of cancer cells and may function as tumor suppressors. One such rare sugar, D-allose, has been shown in human head and neck cancers to induce apoptosis due to competitive inhibition of glucose uptake and lowering of the apoptotic threshold to improve efficacy of the taxane docetaxel (Mitani et al., 2009; Indo et al., 2014). Another rare sugar, L-sorbose, the C-3 epimer of fructose, is taken up and initially phosphorylated similarly to fructose by KHK to produce L-sorbose-1-phosphate (S-1-P). However, unlike fructose, S-1-P inhibits the action of HK at the top of glycolytic flux thereby preventing the synthesis of essential glycolytic metabolites and promoting apoptosis (Xu et al., 2023). In this same vein, mannose, the C-2 epimer of glucose, is also tumor suppressive. Mannose enters the cell through Glut transporters and is phosphorylated by HK to mannose-6-phosphate (M6P). M6P decreases the levels of the anti-apoptotic proteins Mcl-1 and Bcl-X_L_ by inhibiting key glycolytic enzymes including HK and sensitizing cells to the chemotherapeutic cisplatin (Gonzalez et al., 2018). These findings importantly show that it is not necessarily the abundance of sugar but rather the specific sugar source which tips the balance between tumorigenesis and cell death.

## Conclusion and perspectives

As discussed here, apoptosis and metabolism are tightly interwoven cellular processes. The choice of whether or not to activate the cellular suicide program can be dictated by nutrient availability and metabolic pathway engagement. Apoptosis is kept in check through the sequestration of important apoptotic players (e.g., cytochrome *c* in the mitochondria) during normal metabolic flux with robust nutrient sources. Members of the Bcl-2 family of proteins play integral roles in properly trafficking nutrients through their requisite pathways to ensure proper growth and division of the cell. Apoptosis-inducing stimuli alter metabolic flux modifying the function, localization, or both of apoptotic proteins which then either must be blocked or the cell will succumb to this insult. There is inherent difficulty in decoupling the metabolic and death role of apoptotic proteins. The examination of the metabolic role of these proteins through loss of function studies can prove lethal to the cell. Therefore, thorough consideration must be taken to safeguard cell viability when attempting to disentangle the metabolic role from the cell death aspect. Mutations in these proteins which specifically inactivate the apoptotic domain (e.g., altering the BH3 domain of Bcl-2 family proteins) are a useful tool for elucidating the metabolic pathways regulated by the protein of interest. Likewise, in basal conditions or those that do not meet the apoptotic threshold, such as minority MOMP, the metabolic impact of apoptotic regulators could better be considered. This is particularly important given the prevalence of chemotherapeutics which specifically target Bcl-2 family members to kill cancer cells while simultaneously having a metabolic impact on non-malignant tissues (such as immune cells, cancer-associated fibroblasts, etc.). On the other hand, the advent of recently approved cancer therapeutics targeting metabolism may inadvertently trigger pathways that regulate apoptosis (Stine et al., 2022). These alterations to apoptotic signaling may be synergistic or may function to counteract the efficacy of these drugs, but possible knock-on impacts to apoptosis must be considered. Ideally, future ventures to treat cancer will exploit the relationship between apoptosis and metabolism to simultaneously activate the cellular death programs while starving transformed cells of nutrients required for survival.

## References

[R1] Aubrey BJ, Kelly GL, Janic A, Herold MJ, and Strasser A (2018). How does p53 induce apoptosis and how does this relate to p53-mediated tumour suppression? Cell. Death Differ 25, 104–113. doi:10.1038/cdd.2017.169 29149101 PMC5729529

[R2] Autret A, and Martin SJ (2010). Bcl-2 family proteins and mitochondrial fission/fusion dynamics. Cell. Mol. Life Sci 67, 1599–1606. doi:10.1007/s00018-010-0286-x 20143248 PMC11115729

[R3] Bensaad K, Tsuruta A, Selak MA, Vidal MNC, Nakano K, Bartrons R, (2006). TIGAR, a p53-inducible regulator of glycolysis and apoptosis. Cell. 126, 107–120. doi:10.1016/j.cell.2006.05.036 16839880

[R4] Beroukhim R, Mermel CH, Porter D, Wei G, Raychaudhuri S, Donovan J, (2010). The landscape of somatic copy-number alteration across human cancers. Nature 463, 899–905. doi:10.1038/nature08822 20164920 PMC2826709

[R5] Blaineau SV, and Aouacheria A (2009). BCL2DB: Moving ’helix-bundled’ BCL-2 family members to their database. Apoptosis 14, 923–925. doi:10.1007/s10495-009-0376-0 19543976

[R6] Buytaert E, Callewaert G, Vandenheede JR, and Agostinis P (2006). Deficiency in apoptotic effectors BAX and BAK reveals an autophagic cell death pathway initiated by photodamage to the endoplasmic reticulum. Autophagy 2, 238–240. doi:10.4161/auto.2730 16874066

[R7] Cheung EC, Ludwig RL, and Vousden KH (2012). Mitochondrial localization of TIGAR under hypoxia stimulates HK2 and lowers ROS and cell death. Proc. Natl. Acad. Sci. U. S. A 109, 20491–20496. doi:10.1073/pnas.1206530109 23185017 PMC3528527

[R8] Chipuk JE, McStay GP, Bharti A, Kuwana T, Clarke CJ, Siskind LJ , (2012). Sphingolipid metabolism cooperates with BAK and BAX to promote the mitochondrial pathway of apoptosis. Cell. 148, 988–1000. doi:10.1016/j.cell.2012.01.038 22385963 PMC3506012

[R9] Colell A, Ricci JE, Tait S, Milasta S, Maurer U, Bouchier-Hayes L, (2007). GAPDH and autophagy preserve survival after apoptotic cytochrome c release in the absence of caspase activation. Cell. 129, 983–997. doi:10.1016/j.cell.2007.03.045 17540177

[R10] Danial NN, Gramm CF, Scorrano L, Zhang CY, Krauss S, Ranger AM, (2003). BAD and glucokinase reside in a mitochondrial complex that integrates glycolysis and apoptosis. Nature 424, 952–956. doi:10.1038/nature01825 12931191

[R11] Danial NN, Walensky LD, Zhang CY, Choi CS, Fisher JK, Molina AJA, (2008). Dual role of proapoptotic BAD in insulin secretion and beta cell survival. Nat. Med 14, 144–153. doi:10.1038/nm1717 18223655 PMC3918232

[R12] Deng H, Yu F, Chen J, Zhao Y, Xiang J, and Lin A (2008). Phosphorylation of Bad at Thr-201 by JNK1 promotes glycolysis through activation of phosphofructokinase-1. J. Biol. Chem 283, 20754–20760. doi:10.1074/jbc.M800024200 18469002 PMC2475697

[R13] Dixon SJ, Lemberg KM, Lamprecht MR, Skouta R, Zaitsev EM, Gleason CE, (2012). Ferroptosis: An iron-dependent form of nonapoptotic cell death. Cell. 149, 1060–1072. doi:10.1016/j.cell.2012.03.042 22632970 PMC3367386

[R14] Dolgachev V, Farooqui MS, Kulaeva OI, Tainsky MA, Nagy B, Hanada K, (2004). De novo ceramide accumulation due to inhibition of its conversion to complex sphingolipids in apoptotic photosensitized cells. J. Biol. Chem 279, 23238–23249. doi:10.1074/jbc.M311974200 15020599

[R15] Ekoff M, Kaufmann T, Engström M, Motoyama N, Villunger A, Jönsson JI, (2007). The BH3-only protein Puma plays an essential role in cytokine deprivation induced apoptosis of mast cells. Blood 110, 3209–3217. doi:10.1182/blood-2007-02-073957 17634411 PMC2200922

[R16] Elmore S (2007). Apoptosis: A review of programmed cell death. Toxicol. Pathol 35, 495–516. doi:10.1080/01926230701320337 17562483 PMC2117903

[R17] Escudero S, Zaganjor E, Lee S, Mill CP, Morgan AM, Crawford EB, (2018). Dynamic regulation of long-chain fatty acid oxidation by a noncanonical interaction between the MCL-1 BH3 helix and VLCAD. Mol. Cell 69, 729–743.e7. doi:10.1016/j.molcel.2018.02.005 29499131 PMC5916823

[R18] Fawthrop DJ, Boobis AR, and Davies DS (1991). Mechanisms of cell death. Arch. Toxicol 65, 437–444. doi:10.1007/bf01977355 1929863

[R19] Gama V, Swahari V, Schafer J, Kole AJ, Evans A, Huang Y, (2014). The E3 ligase PARC mediates the degradation of cytosolic cytochrome c to promote survival in neurons and cancer cells. Sci. Signal 7, ra67. doi:10.1126/scisignal.2005309 25028717 PMC4182917

[R20] Garufi A, and D’Orazi G (2014). High glucose dephosphorylates serine 46 and inhibits p53 apoptotic activity. J. Exp. Clin. Cancer Res 33, 79. doi:10.1186/s13046-014-0079-4 25260780 PMC4181716

[R21] Giménez-Cassina A, Garcia-Haro L, Choi CS, Osundiji MA, Lane EA, Huang H, (2014). Regulation of hepatic energy metabolism and gluconeogenesis by BAD. Cell. Metab 19, 272–284. doi:10.1016/j.cmet.2013.12.001 24506868 PMC3971904

[R22] Goncalves MD, Lu C, Tutnauer J, Hartman TE, Hwang SK, Murphy CJ, (2019). High-fructose corn syrup enhances intestinal tumor growth in mice. Science 363, 1345–1349. doi:10.1126/science.aat8515 30898933 PMC6487857

[R23] Gonzalez PS, O’Prey J, Cardaci S, Barthet VJA, Sakamaki JI, Beaumatin F, (2018). Mannose impairs tumour growth and enhances chemotherapy. Nature 563, 719–723. doi:10.1038/s41586-018-0729-3 30464341

[R24] Hall PA, Coates PJ, Ansari B, and Hopwood D (1994). Regulation of cell number in the mammalian gastrointestinal tract: The importance of apoptosis. J. Cell. Sci 107 (12), 3569–3577. doi:10.1242/jcs.107.12.3569 7706406

[R25] Hanahan D, and Weinberg RA (2011). Hallmarks of cancer: The next generation. Cell. 144, 646–674. doi:10.1016/j.cell.2011.02.013 21376230

[R26] Hawk MA, Gorsuch CL, Fagan P, Lee C, Kim SE, Hamann JC, (2018). RIPK1-mediated induction of mitophagy compromises the viability of extracellular-matrix-detached cells. Nat. Cell. Biol 20, 272–284. doi:10.1038/s41556-018-0034-2 29459781

[R27] Horn HF, and Vousden KH (2007). Coping with stress: Multiple ways to activate p53. Oncogene 26, 1306–1316. doi:10.1038/sj.onc.1210263 17322916

[R28] Ichim G, Lopez J, Ahmed SU, Muthalagu N, Giampazolias E, Delgado ME, (2015). Limited mitochondrial permeabilization causes DNA damage and genomic instability in the absence of cell death. Mol. Cell 57, 860–872. doi:10.1016/j.molcel.2015.01.018 25702873 PMC4352766

[R29] Indo K, Hoshikawa H, Kamitori K, Yamaguchi F, Mori T, Tokuda M, (2014). Effects of D-allose in combination with docetaxel in human head and neck cancer cells. Int. J. Oncol 45, 2044–2050. doi:10.3892/ijo.2014.2590 25109398

[R30] Jang C, Hui S, Lu W, Cowan AJ, Morscher RJ, Lee G, (2018). The small intestine converts dietary fructose into glucose and organic acids. Cell. Metab 27, 351–361.e3. doi:10.1016/j.cmet.2017.12.016 29414685 PMC6032988

[R31] Jones RG, Plas DR, Kubek S, Buzzai M, Mu J, Xu Y, (2005). AMP-activated protein kinase induces a p53-dependent metabolic checkpoint. Mol. Cell 18, 283–293. doi:10.1016/j.molcel.2005.03.027 15866171

[R32] Kalkavan H, Chen MJ, Crawford JC, Quarato G, Fitzgerald P, Tait SWG, (2022). Sublethal cytochrome c release generates drug-tolerant persister cells. Cell. 185, 3356–3374.e22. doi:10.1016/j.cell.2022.07.025 36055199 PMC9450215

[R33] Kerr JF, Wylie AH, and Currie AR (1972). Apoptosis: A basic biological phenomenon with wide-ranging implications in tissue kinetics. Br. J. Cancer 26, 239–257. doi:10.1038/bjc.1972.33 4561027 PMC2008650

[R34] Kozopas KM, Yang T, Buchan HL, Zhou P, and Craig RW (1993). MCL1, a gene expressed in programmed myeloid cell differentiation, has sequence similarity to BCL2. Proc. Natl. Acad. Sci. U. S. A 90, 3516–3520. doi:10.1073/pnas.90.8.3516 7682708 PMC46331

[R35] Lafont E, Milhas D, Carpentier S, Garcia V, Jin ZX, Umehara H, (2010). Caspase-mediated inhibition of sphingomyelin synthesis is involved in FasL-triggered cell death. Cell. Death Differ 17, 642–654. doi:10.1038/cdd.2009.130 19779494

[R36] Lee SM, Kim JH, Cho EJ, and Youn HD (2009). A nucleocytoplasmic malate dehydrogenase regulates p53 transcriptional activity in response to metabolic stress. Cell. Death Differ 16, 738–748. doi:10.1038/cdd.2009.5 19229245

[R37] Li P, Nijhawan D, Budihardjo I, Srinivasula SM, Ahmad M, Alnemri ES, (1997). Cytochrome c and dATP-dependent formation of apaf-1/caspase-9 complex initiates an apoptotic protease cascade. Cell. 91, 479–489. doi:10.1016/S0092-8674(00)80434-1 9390557

[R38] Liberti MV, and Locasale JW (2016). The warburg effect: How does it benefit cancer cells? Trends Biochem. Sci 41, 211–218. doi:10.1016/j.tibs.2015.12.001 26778478 PMC4783224

[R39] Liu X, Kim CN, Yang J, Jemmerson R, and Wang X (1996). Induction of apoptotic program in cell-free extracts: Requirement for dATP and cytochrome c. Cell. 86, 147–157. doi:10.1016/s0092-8674(00)80085-9 8689682

[R40] Luo W, Hu H, Chang R, Zhong J, Knabel M, O’Meally R, (2011). Pyruvate kinase M2 is a PHD3-stimulated coactivator for hypoxia-inducible factor 1. Cell. 145, 732–744. doi:10.1016/j.cell.2011.03.054 21620138 PMC3130564

[R41] Mai WX, Gosa L, Daniels VW, Ta L, Tsang JE, Higgins B, (2017). Cytoplasmic p53 couples oncogene-driven glucose metabolism to apoptosis and is a therapeutic target in glioblastoma. Nat. Med 23, 1342–1351. doi:10.1038/nm.4418 29035366 PMC5683421

[R42] Mann J, Githaka JM, Buckland TW, Yang N, Montpetit R, Patel N, (2019). Non-canonical BAD activity regulates breast cancer cell and tumor growth via 14-3-3 binding and mitochondrial metabolism. Oncogene 38, 3325–3339. doi:10.1038/s41388-018-0673-6 30635657 PMC6756016

[R43] McArthur K, Whitehead LW, Heddleston JM, Li L, Padman BS, Oorschot V, (2018). BAK/BAX macropores facilitate mitochondrial herniation and mtDNA efflux during apoptosis. Science 359, eaao6047. doi:10.1126/science.aao6047 29472455

[R44] McIlwain DR, Berger T, and Mak TW (2013). Caspase functions in cell death and disease. Cold Spring Harb. Perspect. Biol 5, a008656. doi:10.1101/cshperspect.a008656 23545416 PMC3683896

[R45] Miller M, Craig JW, Drucker WR, and Woodward H Jr. (1956). The metabolism of fructose in man. Yale J. Biol. Med 29, 335–360. 13409929 PMC2603858

[R46] Mitani T, Hoshikawa H, Mori T, Hosokawa T, Tsukamoto I, Yamaguchi F, (2009). Growth inhibition of head and neck carcinomas by D-allose. Head. Neck 31, 1049–1055. doi:10.1002/hed.21070 19340872

[R47] Moyzis AG, Lally NS, Liang W, Najor RH, and Gustafsson Å B (2022). Mcl-1 differentially regulates autophagy in response to changes in energy status and mitochondrial damage. Cells 11, 1469. doi:10.3390/cells11091469 35563775 PMC9102819

[R48] Okawa Y, Hoshino A, Ariyoshi M, Kaimoto S, Tateishi S, Ono K, (2019). Ablation of cardiac TIGAR preserves myocardial energetics and cardiac function in the pressure overload heart failure model. Am. J. Physiol. Heart Circ. Physiol 316, H1366–h1377. doi:10.1152/ajpheart.00395.2018 30901275

[R49] Okorokov AL, and Milner J (1999). An ATP/ADP-dependent molecular switch regulates the stability of p53-DNA complexes. Mol. Cell. Biol 19, 7501–7510. doi:10.1128/mcb.19.11.7501 10523638 PMC84752

[R50] Okoshi R, Ozaki T, Yamamoto H, Ando K, Koida N, Ono S, (2008). Activation of AMP-activated protein kinase induces p53-dependent apoptotic cell death in response to energetic stress. J. Biol. Chem 283, 3979–3987. doi:10.1074/jbc.M705232200 18056705

[R51] Oltval ZN, Milliman CL, and Korsmeyer SJ (1993). Bcl-2 heterodimerizes *in vivo* with a conserved homolog, Bax, that accelerates programmed cell death. Cell. 74, 609–619. doi:10.1016/0092-8674(93)90509-O 8358790

[R52] Opferman JT, Iwasaki H, Ong CC, Suh H, Mizuno S. i., Akashi K, (2005). Obligate role of anti-apoptotic MCL-1 in the survival of hematopoietic stem cells. Science 307, 1101–1104. doi:10.1126/science.1106114 15718471

[R53] Opferman JT, Letai A, Beard C, Sorcinelli MD, Ong CC, and Korsmeyer SJ (2003). Development and maintenance of B and T lymphocytes requires antiapoptotic MCL-1. Nature 426, 671–676. doi:10.1038/nature02067 14668867

[R54] Perciavalle RM, Stewart DP, Koss B, Lynch J, Milasta S, Bathina M, (2012). Anti-apoptotic MCL-1 localizes to the mitochondrial matrix and couples mitochondrial fusion to respiration. Nat. Cell. Biol 14, 575–583. doi:10.1038/ncb2488 22544066 PMC3401947

[R55] Prew MS, Adhikary U, Choi DW, Portero EP, Paulo JA, Gowda P, (2022). MCL-1 is a master regulator of cancer dependency on fatty acid oxidation. Cell. Rep 41, 111445. doi:10.1016/j.celrep.2022.111445 36198266 PMC9933948

[R56] Reynolds JE, Yang T, Qian L, Jenkinson JD, Zhou P, Eastman A, (1994). Mcl-1, a member of the Bcl-2 family, delays apoptosis induced by c-Myc overexpression in Chinese hamster ovary cells. Cancer Res. 54, 6348–6352. 7987827

[R57] Riedl SJ, and Salvesen GS (2007). The apoptosome: Signalling platform of cell death. Nat. Rev. Mol. Cell. Biol 8, 405–413. doi:10.1038/nrm2153 17377525

[R58] Roberts DJ, and Miyamoto S (2015). Hexokinase II integrates energy metabolism and cellular protection: Akting on mitochondria and TORCing to autophagy. Cell. Death Differ 22, 248–257. doi:10.1038/cdd.2014.173 25323588 PMC4291497

[R59] Schafer ZT, and Kornbluth S (2006). The apoptosome: Physiological, developmental, and pathological modes of regulation. Dev. Cell 10, 549–561. doi:10.1016/j.devcel.2006.04.008 16678772

[R60] Schwartzenberg-Bar-Yoseph F, Armoni M, and Karnieli E (2004). The tumor suppressor p53 down-regulates glucose transporters GLUT1 and GLUT4 gene expression. Cancer Res. 64, 2627–2633. doi:10.1158/0008-5472.can-03-0846 15059920

[R61] Separovic D, Hanada K, Maitah MYA, Nagy B, Hang I, Tainsky MA, (2007). Sphingomyelin synthase 1 suppresses ceramide production and apoptosis post-photodamage. Biochem. Biophys. Res. Commun 358, 196–202. doi:10.1016/j.bbrc.2007.04.095 17467659 PMC2701614

[R62] Shi Y, Chen J, Weng C, Chen R, Zheng Y, Chen Q, (2003). Identification of the protein–protein contact site and interaction mode of human VDAC1 with Bcl-2 family proteins. Biochem. Biophysical Res. Commun 305, 989–996. doi:10.1016/S0006-291X(03)00871-4 12767928

[R63] Stine ZE, Schug ZT, Salvino JM, and Dang CV (2022). Targeting cancer metabolism in the era of precision oncology. Nat. Rev. Drug Discov 21, 141–162. doi:10.1038/s41573-021-00339-6 34862480 PMC8641543

[R64] Surget S, Khoury MP, and Bourdon JC (2013). Uncovering the role of p53 splice variants in human malignancy: A clinical perspective. Onco Targets Ther 7, 57–68. doi:10.2147/ott.S53876 24379683 PMC3872270

[R65] Taha TA, Mullen TD, and Obeid LM (2006). A house divided: Ceramide, sphingosine, and sphingosine-1-phosphate in programmed cell death. Biochim. Biophys. Acta 1758, 2027–2036. doi:10.1016/j.bbamem.2006.10.018 17161984 PMC1766198

[R66] Tait SW, Parsons MJ, Llambi F, Bouchier-Hayes L, Connell S, Muñoz-Pinedo C, (2010). Resistance to caspase-independent cell death requires persistence of intact mitochondria. Dev. Cell 18, 802–813. doi:10.1016/j.devcel.2010.03.014 20493813 PMC3004027

[R67] Taylor SR, Ramsamooj S, Liang RJ, Katti A, Pozovskiy R, Vasan N, (2021). Dietary fructose improves intestinal cell survival and nutrient absorption. Nature 597, 263–267. doi:10.1038/s41586-021-03827-2 34408323 PMC8686685

[R68] Vaughn AE, and Deshmukh M (2008). Glucose metabolism inhibits apoptosis in neurons and cancer cells by redox inactivation of cytochrome c. Nat. Cell. Biol 10, 1477–1483. doi:10.1038/ncb1807 19029908 PMC2626347

[R69] von Haefen C, Wieder T, Gillissen B, Stärck L, Graupner V, Dörken B, (2002). Ceramide induces mitochondrial activation and apoptosis via a Bax-dependent pathway in human carcinoma cells. Oncogene 21, 4009–4019. doi:10.1038/sj.onc.1205497 12037683

[R70] Warburg O (1956). On the origin of cancer cells. Science 123, 309–314. doi:10.1126/science.123.3191.309 13298683

[R71] Wei G, Twomey D, Lamb J, Schlis K, Agarwal J, Stam RW, (2006). Gene expression-based chemical genomics identifies rapamycin as a modulator of MCL1 and glucocorticoid resistance. Cancer Cell. 10, 331–342. doi:10.1016/j.ccr.2006.09.006 17010674

[R72] Westphal D, Dewson G, Czabotar PE, and Kluck RM (2011). Molecular biology of Bax and Bak activation and action. Biochimica Biophysica Acta (BBA) - Mol. Cell. Res 1813, 521–531. doi:10.1016/j.bbamcr.2010.12.019 21195116

[R73] Wuillème-Toumi S, Robillard N, Gomez P, Moreau P, Le Gouill S, Avet-Loiseau H, (2005). Mcl-1 is overexpressed in multiple myeloma and associated with relapse and shorter survival. Leukemia 19, 1248–1252. doi:10.1038/sj.leu.2403784 15902294

[R74] Xu HL, Zhou X, Chen S, Xu S, Li Z, Nakanishi H, (2023). Rare sugar L-sorbose exerts antitumor activity by impairing glucose metabolism. Commun. Biol 6, 259. doi:10.1038/s42003-023-04638-z 36906698 PMC10008635

[R75] Xue D, and Robert Horvitz H (1995). Inhibition of the *Caenorhabditis elegans* cell-death protease CED-3 by a CED-3 cleavage site in baculovirus p35 protein. Nature 377, 248–251. doi:10.1038/377248a0 7675111

[R76] Yang E, Zha J, Jockel J, Boise LH, Thompson CB, and Korsmeyer SJ (1995). Bad, a heterodimeric partner for Bcl-XL and Bcl-2, displaces Bax and promotes cell death. Cell. 80, 285–291. doi:10.1016/0092-8674(95)90411-5 7834748

[R77] Yu H-P, Xie JM, Li B, Sun YH, Gao QG, Ding ZH, (2015). TIGAR regulates DNA damage and repair through pentosephosphate pathway and Cdk5-ATM pathway. Sci. Rep 5, 9853. doi:10.1038/srep09853 25928429 PMC4415581

[R78] Zhao Y, Coloff JL, Ferguson EC, Jacobs SR, Cui K, and Rathmell JC (2008). Glucose metabolism attenuates p53 and Puma-dependent cell death upon growth factor deprivation. J. Biol. Chem 283, 36344–36353. doi:10.1074/jbc.M803580200 18990690 PMC2606014

[R79] Zhou P, Qian L, Kozopas KM, and Craig RW (1997). Mcl-1, a Bcl-2 family member, delays the death of hematopoietic cells under a variety of apoptosis-inducing conditions. Blood 89, 630–643. doi:10.1182/blood.v89.2.630 9002967

